# Rectal Adenocarcinoma With Pagetoid Spread: A Novel Entity

**DOI:** 10.1155/2024/4952952

**Published:** 2024-10-21

**Authors:** Lefika Bathobakae, Pasha Shenasan, Aakash Trivedi, Ruhin Yuridullah, Sohail Qayyum, Abraham El-Sedfy

**Affiliations:** ^1^Internal Medicine, St. Joseph's University Medical Center, Paterson, New Jersey, USA; ^2^General Surgery, St. Joseph's University Medical Center, Paterson, New Jersey, USA; ^3^Gastroenterology & Hepatology, St. Joseph's University Medical Center, Paterson, New Jersey, USA; ^4^Pathology & Lab Medicine, St. Joseph's University Medical Center, Paterson, New Jersey, USA; ^5^Colorectal Surgery, St. Joseph's University Medical Center, Paterson, New Jersey, USA

**Keywords:** Paget's disease, pagetoid spread, perianal Paget's disease, rectal adenocarcinoma, secondary Paget's disease, total neoadjuvant therapy

## Abstract

Perianal Paget's disease (PPD) is a rare skin adenocarcinoma that arises in the apocrine glands of the perianal region. It is often misdiagnosed as eczema, leukoplakia, squamous cell carcinoma, Bowen's disease, lichen planus, or condylomata acuminata. We report a case of a 63-year-old male who presented to the emergency room (ER) for evaluation of an anal mass that had persisted over 6 months. The patient was found to have a rectal adenocarcinoma with pagetoid spread and underwent neoadjuvant chemoradiation with symptom improvement. However, the patient declined further chemotherapy and the planned abdominal perineal resection with reconstruction, as it would require a permanent colostomy.

## 1. Introduction

Paget's disease presents as a pruritic ulcerative weeping skin lesion arising from apocrine glands [1, 2]. Beyond the areola/nipple complex, Paget's disease can be found in the axilla, penis, scrotum, vulva, and the perianal region [2]. First described in the French literature by Darier and Couillard in 1893 [2, 3], Perianal Paget's Disease (PPD) is a rare variant of Extramammary Paget's disease (EMPD) [4]. PPD can present with no associated malignancy (primary) or be reflective of an underlying gastrointestinal malignancy (secondary) [1]. An underlying invasive carcinoma portends a poor prognosis for PPD, therefore, a thorough search for an underlying primary tumor is crucial before initiating any treatment [1]. PPD accounts for less than 1% of all anal pathologies [4], and because of its rarity, there is scant data on its histogenesis and management. Herein, we describe a rare case of rectal adenocarcinoma with pagetoid spread.

## 2. Case Report

A 63-year-old male with a remote history of open appendectomy presented to the ER for the evaluation of an anal mass of 6 months. The patient also complained of anal pain, blood-streaked stools, pruritus, tenesmus, and dyschezia for the same duration. The perianal pain improved with acetaminophen. The patient also reported a 15 pounds unintentional weight loss in the 3 months preceding the ER visit. He denied nausea, vomiting, early satiety, abdominal pain, low appetite, constipation, diarrhea, or fecal incontinence. The patient denied any personal or family history of colon cancer or inflammatory bowel disease. He denied a prior colonoscopy or engaging in anoreceptive sexual intercourse.

**Figure 1:**
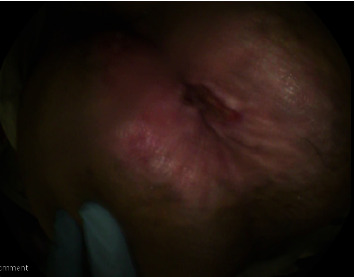
Pink and excoriated skin around the anal verge consistent with a pagetoid spread.

In the ER, the patient was afebrile and vital signs were notable for tachycardia at 104 beats per minute. The patient was thin-appearing and in distress due to pain. Perianal examination revealed pink and excoriated skin around the anal verge (Figure 1). No evidence of fistulas, fissures, abscesses, discharge, or active bleeding was found. Anal examination was deferred due to pain and discomfort. The rest of the physical examination was unremarkable.

**Figure 2:**
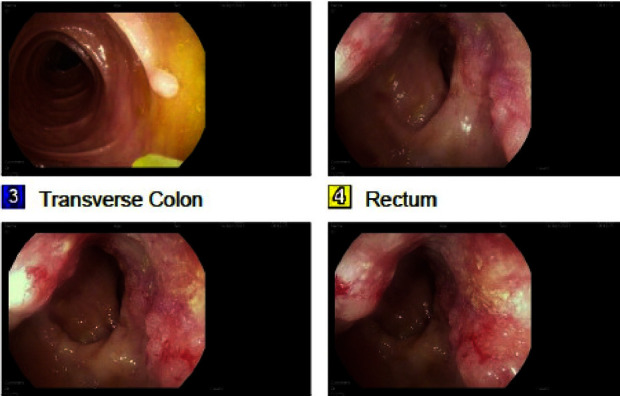
Endoscopy image showing a 7 mm polyp in the distal transverse colon and frond-like/villous, infiltrative, and ulcerated, nonobstructing large mass in the anus extending proximally.

Blood work showed a white blood cell count of 7.0 × 10^3^/mm^3^ and hemoglobin of 14.4 g/dL. The rest of the cell lines and chemistry were unremarkable. Computed tomography (CT) scan of the abdomen and pelvis showed mildly prominent stools in the colon without obstruction. Anorectal examination under anesthesia revealed an infiltrative, ulcerated, and nonobstructing anorectal mass. This was biopsied using cold forceps for histology from the 12, 10 cm, and anorectal regions. The overlying anal skin showed extensive secondary Paget's disease. Colonoscopy showed a 6 cm frond-like, villous, ulcerated, and nonobstructing anal mass involving 2/3 of the lumen circumference, extending proximally (Figure 2). A 7 mm transverse colon polyp was resected, and the pathology showed mild reactive crypt hyperplasia that was negative for adenomatous dysplasia or neoplastic processes.

**Figure 3:**
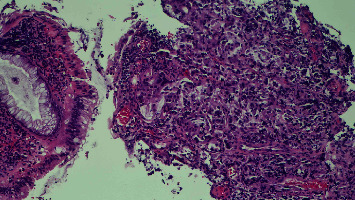
Histology slide showing minute fragments of hyperplastic rectal mucosa and a tiny fragment demonstrating atypical back-to-back glandular proliferation with focal complex architecture and clusters of atypical cells in lamina propria consistent with adenocarcinoma.

**Figure 4:**
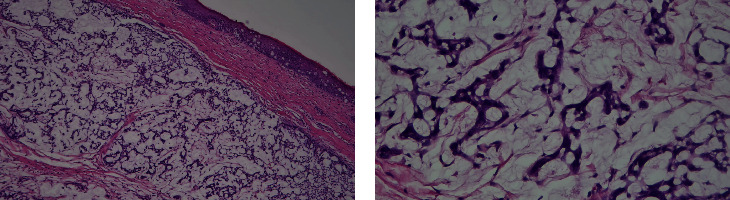
Histology slides showing large, pale staining-to-clear intraepidermal neoplastic cells containing abundant mucin, arranged in gland-like formations, consistent with Paget's disease.

The final pathology of the resected mass confirmed mucinous adenocarcinoma with areas of squamous differentiation arising from the rectum and extending to the anus (Figures 3 and 4). Positron emission tomography (PET) scan showed a localized lesion in the anorectal region without metastatic disease. The patient was diagnosed with T3N0M0 rectal adenocarcinoma with pagetoid spread.

The patient's initial treatment plan included neoadjuvant chemoradiation therapy, surgical resection, and adjuvant chemotherapy according to the National Comprehensive Cancer Network (NCCN) guidelines. The patient's pagetoid spread was included in the radiation field and it shrunk. After completion of neoadjuvant chemoradiation therapy, the patient's treatment team discussed the option of proceeding with total neoadjuvant therapy (TNT), followed by restaging and resection. The patient declined further treatment, including a planned abdominal perineal resection with cutaneous flap reconstruction, citing concerns over permanent colostomy. Despite extensive counseling on his condition and alternative nonsurgical treatment, the patient declined further treatment, and his autonomy was respected. The patient still experienced occasional pruritus and pain in the intergluteal and anal areas, which were controlled using sitz baths. Mismatch repair gene (MMR) testing on the tumor (as per NCCN guidelines) had a low probability of microsatellite instability; thus, the tumor did not appear to be associated with Lynch syndrome.

## 3. Discussion

Paget's disease is a rare skin adenocarcinoma of the breast tissue, initially reported by Sir James Paget in 1874 [1, 5]. Paget's disease has been observed in extramammary organs, including the vulva, scrotum, penile skin, axilla, and buttocks, nose, external ear, and umbilicus [1–3, 5]. EMPD is more prevalent among white-skinned women aged 50–80 years [1, 6, 7]. PPD is a subtype of EMPD that arises in apocrine glands in the perianal region [3]. Primary PPD (p-PPD) arises from the epidermis or epidermal appendages, including sweat glands [8], in the perirectal area. Secondary PPD (s-PPD), also known as pagetoid spread, is a metastatic tumor that originates from an underlying gastrointestinal or urological neoplasm [8].

To date, less than 200 cases of PPD have been reported in the literature [1, 4, 7], with only a fraction associated with an underlying gastrointestinal neoplasm. Because of its vague presentation and indolent onset, PPD can be misdiagnosed as eczema, leukoplakia, squamous cell carcinoma, Bowen's disease, lichen planus, or condylomata acuminata [5]. Kim et al. [4] reported a case of PPD in an elderly Korean male who was misdiagnosed with treatment refractory-eczema after failing a treatment of topical steroids and anti-histamines. Similarly, Jabir et al. [6] described a case of PPD that was misdiagnosed as dermatitis. Like our patient, most patients with PPD present with perirectal pruritus as the initial symptom [3]. In case of underlying colorectal malignancy, patients may also exhibit rectal bleeding, perianal soreness, tenesmus, changes in stool caliber, mucoid discharge, or diarrhea [5].

PPD manifests as crusts, scales, ulcers, verrucous, or hypopigmented lesions in the perianal area [4, 5]. The affected skin is usually soft to touch during palpation, but the presence of induration may suggest malignant invasion [5]. A digital rectal examination can help estimate the intra-anal extent of the disease [5]. PPD can be seen on visual inspection of the perianal area, but a definitive diagnosis can only be made on biopsy. The histopathology of PPD shows Paget cells in the epidermis with a characteristic clear cytoplasm with large polymorphic nuclei [8]. Paget cells stain well with periodic acid-Schiff (PAS) because of their high mucin content [7, 9]. Immunohistochemistry (IHS) studies help distinguish p-PPD from the pagetoid spread [10]. Tumor markers, Cytokeratin 7 (CK7), CK20, and caudal-related homeobox gene nuclear transcription factor (CDX)2, are commonly seen in s-PPD, whereas gross cystic disease fluid protein (GCDFP)15 is pathognomonic for p-PPD [8]. Our patient's IHS profile was positive for CDX2, CK20, CK7, and p40, consistent with s-PPD.

Surgical resection with a margin of 1–3 cm is the gold standard for the management of PPD [2, 6]. Options range from wide local excision to abdominoperineal resection with or without radiation therapy [2]. For the most part, the extent of the resection depends on the staging of the anorectal malignancy [2]. Some patients with s-PPD may require a colostomy after the wide local resection [9]. Skin and or muscle reconstruction is usually performed to fix the tissue defects after the resection [4]. Our patient completed neoadjuvant chemoradiation therapy, but refused further treatment including the proposed abdominoperineal resection and cutaneous flap reconstruction surgery.

Various nonsurgical modalities are available for PPD treatment, including both topical and systemic chemotherapy, radiotherapy, and photodynamic therapy (PTD) [3, 6]. These modalities are appropriate for elderly patients who are not suitable for general anesthesia, those with significant metastatic disease burden, patients who refuse mutilating surgery, and those with occult and multifocal disease [6, 7]. Although data is limited to sporadic case reports, PTD has proved effective against noninvasive PPD, often achieving a complete cure. [11, 12] Pain and irritation are the most documented side effects of this modality. Ablative laser therapy with carbon dioxide or holmium is another therapeutic option in the PPD armamentarium with comparable efficacy. Given its high rates of disease recurrence, ablative therapy is less employed compared to other treatment options. Laser treatment is associated with scarring, risk of bleeding, and delayed healing [13].

Topical agents such as bleomycin, imiquimod, and 5 fluorouracil can offer symptomatic relief and, in some cases, reduce tumor burden before surgical excision [6]. In a systematic review of 24 studies comprising 233 EMPD patients, Mayo-Martínez et al. [14] found that topical imiquimod therapy had a response rate of 67% and a complete response rate of 48%. In these cases, imiquimod cream was used as adjuvant or neoadjuvant therapy or for recurrent disease [14]. Imiquimod cream is applied daily to twice weekly over 2-56 weeks. Interestingly, no correlation has been found between the duration of therapy and the complete response rate [13]. The most common side effects include localized tenderness, irritation, edema, erosions, and erythema [13, 14]. Systemic chemotherapy with trastuzumab and paclitaxel has been found to be effective in metastatic EMPD, where the overexpression of human epidermal growth factor receptor 2 protein leads to deep dermal invasion and metastatic spread of the tumor [6]. There are mixed results regarding the routine use of radiation therapy as an adjunct to surgical resection. Perianal radiotherapy is associated with acute dermatitis, skin atrophy, cystitis, proctitis, and enteritis [6, 7]. Patients with EMPD require long-term observation to prevent disease recurrence and the development of associated malignancies [3]. The interval testing includes an annual punch biopsy from the margins of the old perianal lesion, and a surveillance colonoscopy every 2 years [3].

## 4. Conclusion

Paget's disease manifests as pruritic ulcerative weeping skin lesions originating from the apocrine glands. In addition to the areola/nipple complex, Paget's disease may occur in the axilla, penis, scrotum, vulva, and the perianal region. PPD is a less common form of EMPD that is often misdiagnosed as eczema, leukoplakia, squamous cell carcinoma, Bowen's disease, lichen planus, or condylomata acuminata. Although infrequent, PPD should be considered in the differential diagnosis of perianal lesions to avoid misdiagnosis and delay in treatment. Herein, we present a unique case of a 63-year-old male who presented with a 6-month history of a palpable anal mass. On further investigation, the patient was diagnosed with rectal adenocarcinoma with s-PPD. The patient completed a part of the chemoradiation therapy but declined further treatment due to concerns for a permanent colostomy. The patient also refused alternative nonsurgical therapies despite extensive counseling.

## Data Availability

Further inquiries can be directed to the corresponding author.
